# Efficiency of insect-proof net tunnels in reducing virus-related seed degeneration in sweet potato

**DOI:** 10.1111/ppa.13069

**Published:** 2019-07-22

**Authors:** K. O. Ogero, J. F. Kreuze, M. A. McEwan, N. D. Luambano, H. Bachwenkizi, K. A. Garrett, K. F. Andersen, S. Thomas-Sharma, R. A. A. van der Vlugt

**Affiliations:** a International Potato Center (CIP), c/o Tanzania Agricultural Research Institute (TARI) –Ukiriguru, PO Box 1433, Mwanza, Tanzania; b Plant Research International, Wageningen University and Research (WUR), PO Box 16,6700 AAWageningen, Netherlands; c International Potato Center (CIP), Avenida La Molina 1895, Apartado 1558, Lima, Peru; d International Potato Center, Regional Office for Sub-Saharan Africa (CIP – SSA), ILRI Campus, Old Naivasha Road, PO Box 25171-00603, Nairobi, Kenya; e TARI – Mikocheni, PO Box 6226, Dar Es Salaam, Tanzania; f Plant Pathology Department, University of Florida, Gainesville, FL 32611-0680; g Institute for Sustainable Food Systems, University of Florida, Gainesville, FL 32611-0680; h Emerging Pathogens Institute, University of Florida, Gainesville, FL 32611-0680; i Department of Plant Pathology and Crop Physiology, Louisiana State University Agricultural Center, Baton Rouge, LA 70803-1720, USA

**Keywords:** farmer-multiplier, modelling, net tunnels, seed, sweet potato, virus-related degeneration

## Abstract

Virus-related degeneration constrains production of quality sweet potato seed, especially under open field conditions. Once in the open, virus-indexed seed is prone to virus infection leading to decline in performance. Insect-proof net tunnels have been proven to reduce virus infection under researcher management. However, their effectiveness under farmer-multiplier management is not known. This study investigated the ability of net tunnels to reduce degeneration in sweet potato under farmer-multiplier management. Infection and degeneration were assessed for two cultivars, Kabode and Polista, grown in net tunnels and open fields at two sites with varying virus pressures. There was zero virus incidence at both sites during the first five generations. Sweet potato feathery mottle virus and sweet potato chlorotic stunt virus were present in the last three generations, occurring singly or in combination to form sweet potato virus disease. Virus infection increased successively, with higher incidences recorded at the high virus pressure site. Seed degeneration modelling illustrated that for both varieties, degeneration was reduced by the maintenance of vines under net tunnel conditions. The time series of likely degeneration based on a generic model of yield loss suggested that, under the conditions experienced during the experimental period, infection and losses within the net tunnels would be limited. By comparison, in the open field most of the yield could be lost after a small number of generations without the input of seed with lower disease incidence. Adopting the technology at the farmer-multiplier level can increase availability of clean seed, particularly in high virus pressure areas.

## Introduction

Sweet potato is an important staple and co-staple food crop in Africa, and orange-fleshed sweet potato varieties are a rich source of vitamin A, especially important for infants and young children. The crop is particularly important in Lake Zone, Tanzania (regions around the Lake Victoria basin) where approximately 15 million inhabitants, a third of Tanzania’s population, live (Lembris & Walsh, 2012). However, sweet potato yields are heavily reduced by viruses, which are carried from generation to generation through recycling of infected cuttings (Gibson & Kreuze, 2015). The most common viruses found infecting sweet potato in Africa are sweet potato feathery mottle virus (SPFMV) and sweet potato chlorotic stunt virus (SPCSV) (Loebenstein, 2015). SPFMV is a potyvirus transmitted nonpersistently by aphids (Karyeija *et al*., 1998). It can combine with SPCSV, a crinivirus transmitted semipersistently by whiteflies (Schaefers & Terry, 1976), to cause sweet potato virus disease (Mukasa *et al*., 2006). Individually, SPFMV can cause negligible to 40% loss of root yields (Milgram *et al*., 1996; Adikini *et al*., 2016) whereas SPCSV by itself can lead to 50% or more yield reduction (Loebenstein, 2012). However, in combination, the two viruses have a synergistic effect and cause sweet potato virus disease (SPVD) which is the main ‘virus disease’ affecting the crop, often leading to 56–98% yield losses (Ngeve & Bouwkamp, 1991; Ndunguru *et al*., 2009). Of the two, SPCSV is the most important because it leads to the plant losing any resistance to SPFMV (Nwankwo & Opara, 2015). In addition, a group of geminiviruses, collectively known as sweepoviruses, is increasingly being recognized as damaging and common worldwide, including Africa (Rey *et al*., 2012). Managing such a complex set of viruses is challenging, especially as many of them show no, or only minor transient symptoms when infecting sweet potato alone, making it difficult to identify infected plants (Valderde *et al*., 2007). This is especially true for most African cultivars. Corresponding low titres in plants make their detection by serological methods equally challenging.

There are three major alternatives in managing viruses in sweet potato: (i) deploying resistant cultivars, (ii) using clean (virus-tested) seed, and (iii) employing proper on-farm management practices. Deployment of resistant cultivars is viewed as the most effective strategy in SPVD management (Maule *et al*., 2007). (In this context, the term ‘seed’ refers to quality (virus-indexed) cuttings or storage roots that have been selected for use in generating new plants; it does not refer to just ‘any vine’, or botanical seed that is used for breeding.) However, whereas landraces and cultivars with higher levels of resistance to SPVD do exist, no immunity to the disease exists, and depending on the virus pressure in the environment all genotypes can become infected in the field (Gibson & Kreuze, 2015). The complex genetics of virus resistance in a hexaploid outcrossing crop additionally make progress through breeding slow (Stephan *et al*., 2013). The frequency of obtaining SPVD-resistant geno-types in the Ugandan screening schemes at Namulonge is typically less than 0.2% (Mwanga *et al*., 2002). This has presented a major bottleneck for introducing new orange-fleshed sweet potato varieties high in vitamin A into East Africa, especially in high virus pressure areas.

Complementary to the use of genetic resistance could be the production and use of healthy planting material, largely free of viruses. In developed countries, this is most commonly achieved through formal and centralized certified seed production schemes. However, producing such planting material is expensive. And although this may work well in countries where sweet potato is grown as a cash crop and large-scale farmers can make the investments necessary to obtain such planting material, this has not been economically feasible for smallholder farmers producing mostly for subsistence. On-farm management strategies such as roguing and positive selection for clean seed are therefore important. Roguing is the removal of plants that have virus symptoms whereas positive selection is selection of vigorous healthy-looking plants as planting material/seed for the next season (Muturi *et al*., 2007). The two approaches reduce virus inoculum and hence disease incidence. A study conducted in Uganda showed that removal of diseased plants within 1 month after planting reduced the spread of SPVD (Gibson *et al*., 2004). Selection of planting material from symptomless plants has also been reported to reduce virus incidence (Aritua *et al*., 2003). However, these methods require good farmer knowledge about disease identification. Alternatives that could enable farmers, or specialized local vine multipliers, to maintain a high sanitary status of planting material at low cost and minimum technical input exist. One such technology is a low-cost insect-proof net tunnel that can be constructed from locally sourced materials (Schulte-Geldermann *et al*., 2012). This technology enables farmers to maintain a nuclear stock of high phytosanitary status vines, by protecting them against the virus vectors such as whiteflies and aphids (Loebenstein, 2015). Vines produced in the net tunnels can be harvested and used either directly, or after one or more cycles of field multiplication for root production and/or sale as quality planting material (Ogero *et al*., 2015). However, it is important to know how well net tunnels perform in maintaining the phytosanitary status of sweet potato vines under farmer-multiplier management.

This study sought to determine the rate of virus infection and related degeneration in sweet potato planting material maintained in net tunnels and open fields under farmer-multiplier management. In an integrated seed health strategy, choice of seed and on-farm management should be considered together to optimize management of seed degeneration in vegetatively propagated crops (Thomas-Sharma *et al*., 2016). This study used the observed rates of seed degeneration in the model by Thomas-Sharma *et al*. (2017) to evaluate the likely long-term patterns of degeneration and when purchase of quality-declared seed would be motivated, as a function of management decisions. Once estimates of the rate of infection for particular system combinations – variety, location and management – are available, these can be used in scenario modelling to understand economic thresholds for purchase of quality-declared seed (Thomas-Sharma *et al*., 2017). The models include the level of host resistance, environmental conduciveness, vector management, roguing, the amount of previously infected seed material, and rates of reversion to healthy status. Reversion in virus infection is the ability of infected plants to become almost virus-free after several seasons of cultivation and has been demonstrated in several sweet potato cultivars (Gibson & Kreuze, 2015). Modelling can be used to evaluate potential economic thresholds for seed replacement before yield loss becomes too limiting. Simulation models can extrapolate results to larger areas when multi-year and multi-location trials are cost-prohibitive.

## Materials and methods

### Location and varieties used

This research was conducted at two sites, Mwasonge (02°40′13″ S, 32°54′45″E) and Nyasenga (02°39′40.1″S, 32°44′30.6″E) villages, in Mwanza Region, Lake Zone, Tanzania. Mwasonge is a high virus pressure area owing to high intensity of sweet potato production, whereas Nyasenga is a low virus pressure area due to limited sweet potato cultivation in the area. High cultivation of sweet potato at Mwasonge also translates to higher vector populations due to year-round availability of host plants as compared to Nyasenga. Two sweet potato varieties, Kabode and Polista, were used. Polista is a cream-fleshed local variety, and Kabode is an orange-fleshed Ugandan-bred variety also known as NASPOT 10.

### Experimental set up

**Figure 1:**
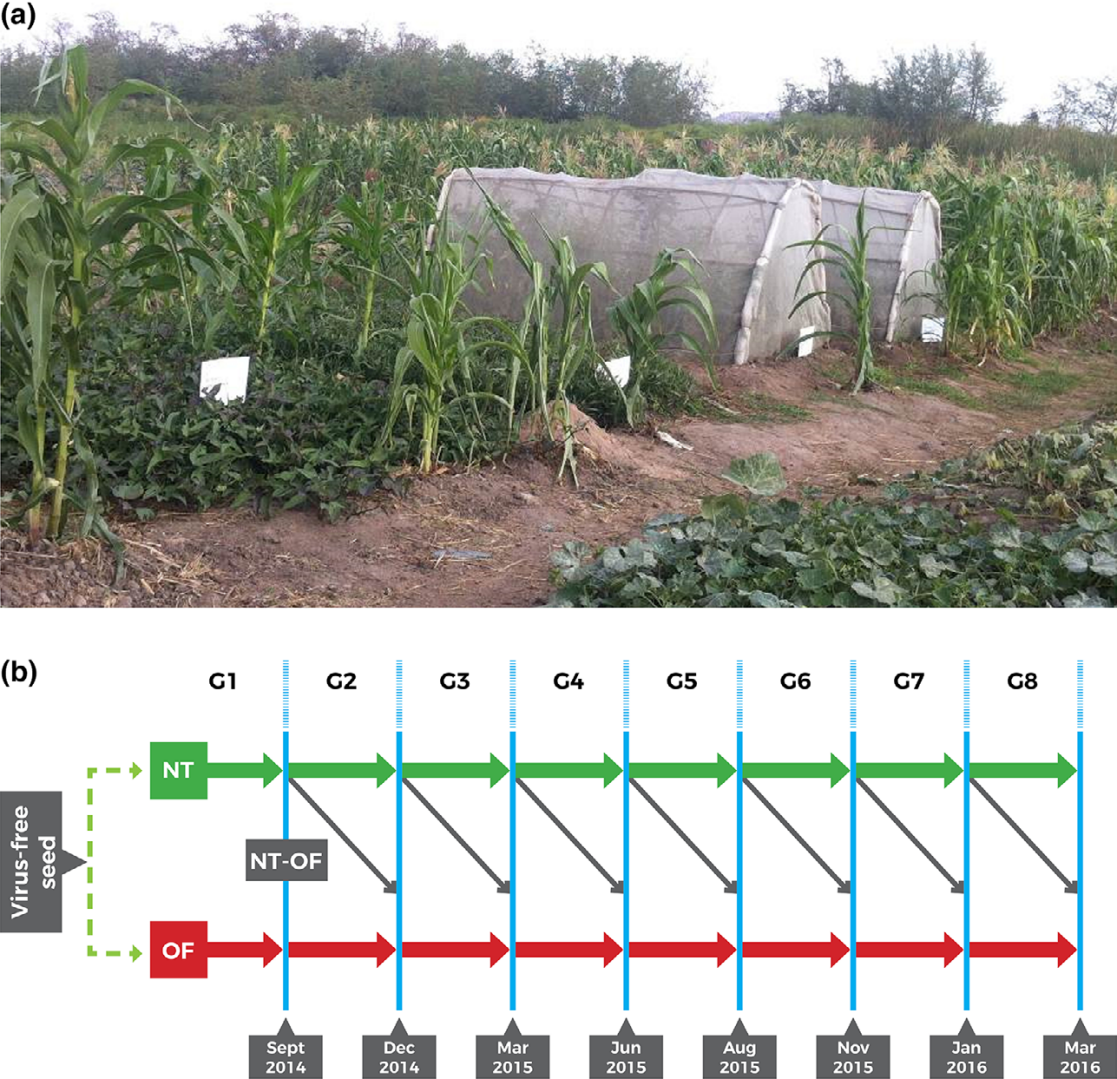
(a) Net tunnels (right) and open fields (left) at the high virus pressure site (Mwasonge). (b) The growing cycles of the experiments: the green line is the intervention (net tunnels, NT) and the red line is the control (open fields, OF). Black arrows indicate vines harvested from the net tunnels and multiplied once in the open field (NTOF); blue vertical lines indicate points of leaf sampling. G1–G8, generations 1–8.

Three-node virus-indexed cuttings were planted in two net tunnels and two open beds (control) at each site – one net tunnel and open bed per variety (Fig. 1a).

Each net tunnel and open bed measured 3 × 1.7 m and had 270 cuttings planted in nine rows at a spacing of 20 × 10 cm. After every 60–80 days, vines were harvested from the net tunnels and planted in the open for a single generation, then destroyed (black arrows in Fig. 1b). The control plants were maintained in the open during the entire period of the study. Visual assessment for virus symptoms was done regularly and any plant that seemed infected removed. Leaf sampling was done after every 60–80 days. Thirty samples were randomly collected from each bed. One leaf from the middle part of each plant was collected into a coffee-filter and put in a zip-lock bag containing 100 g silica gel. Sample collection was done eight times between June 2014 and March 2016 (21 months).

### Environmental virus pressure and weather conditions

Local environmental virus pressure at the multiplication sites was assessed eight times by visually surveying fields and weeds in a radius of 250 m surrounding each plot. Whitefly abundance was recorded by counting for 10 s on 10 leaves from 10 different plants and recording the number. Onset HOBO data loggers were used to record daily weather data (rainfall, relative humidity and temperature).

### Testing viruses present in the environment at initiation of experiment

At the time of establishment of the experiment, samples were taken from nearby sweet potato plots to determine the viruses present in the environment. This could be done at Mwasonge only, because no sweet potato plots were found near the Nyasenga site. Samples were bulked together and subjected to small RNA sequencing assembly to identify any viruses infecting them, as described previously (Kreuze *et al*., 2009).

### Testing for viruses on samples collected from experimental plots and determination of virus incidence

Leaf samples were analysed in bulks of five, and each bulk was screened for SPLCV, potyviruses (SPFMV, sweet potato virus G, sweet potato virus 2 and sweet potato virus C), and SPCSV using PCR, multiplex reverse transcription PCR (RT-PCR), and reverse transcription quantitative real-time PCR (RT-qPCR), respectively. If a bulk was positive, each sample was analysed separately to determine which one was positive. RNA was extracted using a CTAB method with minor modifications in reagents used (Lodhi *et al*., 1994). Extracted total RNA was treated with DNase I to remove DNA before measuring purity and concentration of RNA using a NanoDrop 2000 spectrophotometer (Thermo Scientific). Additionally, total RNA was run on a gel containing 1% agarose (Invitrogen) and viewed on a gel documentation machine (Biodoc-H Imaging system with benchtop UV transilluminator) for RNA integrity. Thereafter, total RNA was diluted with nuclease-free water to end up with an estimated concentration of 0.025 ng μL^−1^.

#### qPCR for SPCSV

The reaction mix for one reaction of qPCR was prepared by mixing 12.5 μLof 2× TaqMan universal master mix (Applied Biosystems), 0.75 μL of each forward (F) and reverse (R) primers (10 μM each; Table 1), 8 μL of nuclease-free water, 0.5 μL of RevertAid reverse transcriptase (Thermo Fisher Scientific), diluted 1/100 (2 U μL^−1^), 0.125 μL of F and R COX (10 μM) (Applied Biosystems), 0.25 μL of probe (5 μM; Applied Biosystems), 0.25 μL of probe for COX (5 μM), and 1 μL of total RNA. Real-time PCR machines (Stratagene Mx3000P; Agilent) were programmed for the following conditions: 30 min at 42 °C, 10 min at 95 °C; followed by 40 cycles of 15 s at 95 °C and 1 min at 60 °C. Results were viewed and recorded using Mx3000P software (Stratagene).

**Table 1:** Primers and probes used for PCR assays.

Primer	Sequence (5′–3′)	Expected size (bp)
SPCSV		
SPCSV-Uni-E-F	CGGAGTTTATTCCCACYTGTYT	
SPCSV-Uni-E-R	GGGCAGCCYCACCAA	
COX-F	CGTCGCATTCCAGATTATCCA	
COX-R	CAACTACGGATATATAAGAGCCAAAACTG	
SPCSV-Uni-E-P	[FAM]-TCTGTCACGGCTACAGGCGACGTG-[TAMRA]	
COX-P	[VIC]-TGCTTACGCTGGATGGAATGCCCT-[TAMRA]	
Potyviruses (SPFMV, SPVC, SPVG, SPV2)		
SPG-F	GTATGAAGACTCTCTGA CAAATTTTG	1191
SPC-F	GTGAGAAAYCTATGCGCTCTGTT	836
SPF-F	GGATTAYGGTGTTGACGACACA	589
SP2-F	CGTACATTGAAAAGAGAAACAGGATA	369
SPFCG2-R	TCGGGACTGAARGAYACGAATTTAA	
Begomoviruses, including sweet potato leaf curl virus (SPLCV)		
SPG1	CCCCKGTGCGWRAATCCAT	912
SPG2	ATCCVAAYWTYCAGGG AGCTAA	

#### Multiplex reverse transcription (RT) PCR for potyviruses

Multiplex RT-PCR for sweet potato potyviruses was performed as described by Li *et al*. (2012) with minor modifications. Total RNA with reverse primer SPFCG2-R (Table 1) was denatured at 65 °C for 10 min and cDNA synthesized using 5× RT buffer, 0.1 M DTT, 10 mM dNTPs, RNAse Out inhibitor 40 U μL ^−1^ and M-MLV 200 U μL^−1^, and the reaction was incubated at 40 °C for 60 min, 95 °C for 5 min. Reaction mix for PCR was prepared using 5× Dream Taq buffer (Invitrogen), 25 mM MgCl_2_,10 mM dNTPs (Invitrogen), primers SPG-F (2.5 μL), SPC-F (0.4 μL), SPF-F (2 μL), SP2-F (0.2 μL) and SPFCGG2-R (2 μL), each at a concentration of 10 μM, Dream Taq 5 U μL ^−1^ and cDNA. The mixture was incubated in a thermal cycler machine (GenAmp PCR system 9700; Applied Biosystems) using the following conditions: 94 °C for 2 min, 94 °C for 30 s; then 35 cycles of 60 °C for 30 s, 72 °C for 1 min 20 s, 72 °C for 10 min. The gel was prepared using 1.2% agarose in 1× TAE buffer and ethidium bromide was used for staining. This was run at 200 V for 30 min and the product viewed and recorded using a gel documentation machine.

#### PCR for SPLCV

The reaction mix was prepared using 5× Dream Taq buffer, 25 mM MgCl_2_,10 mM dNTPs, primers SPG1 (0.5 μL) and SPG2 (0.75 μL) at 10 μM concentration described by Li *et al*. (2004) (Table 1), Dream Taq 5 U μL^−1^ and DNA template. The reaction mix was incubated in a thermal cycler machine (GenAmp PCR System 9700) using the following conditions (touch down): 94 °C for 40 s, 72 °C for 30 s, 72 °C for 90 s; for 11 cycles (*n* – 1 °C per cycle); then 94 °C for 40 s, 60 °C for 40 s, 72 °C for 92 s for 24 cycles; followed by 72 °C for 10 min. The gel was prepared, and products viewed as in RT-PCR above.

*Calculating virus incidence* The number of samples testing positive for various viruses was established through counting and virus incidence at 95% confidence interval calculated as follows:

$$\frac{\text{infected samples}}{\text{sample size}}\pm \Phi^{-1}\left(0.0975\sqrt{\frac{\text{infected samples/sample size}*(1-\text{infected samples/sample size})*\left(\text{population size}-\text{sample size}\right)}{\left(\text{sample size}*\left(\text{population size}-1\right)\right)}}\right)$$

### Modelling of seed degeneration

To model the likely influence of the management components evaluated in this study on seed degeneration over the course of 10 seasons, virus incidence data were used to estimate parameters in the model from Thomas-Sharma *et al*. (2017). Four treatment combinations were compared using data from Mwasonge, the ‘high virus’ location. Treatments where cv. Polista and cv. Kabode were grown in the net tunnel, with seed continuously replaced from the net tunnel for each season, were compared with treatments where seed for these two cultivars was continuously grown and obtained from the open field. Virus incidence data collected for the last three generations (generations 6, 7 and 8) for each of these four treatment combinations were used to parameterize the proportional change in infection due to the combined effect of environment, host and management (Thomas-Sharma *et al*., 2017). Simulation experiments were carried out in the R programming environment (R Core Team, 2018) using the custom package seedHealth (https://www.garrettlab.com/software/). Each parameter combination was evaluated in 1000 simulations, over 10 generations. Details of parameters used in simulations for each of the four scenarios are given in Table S1. Note that this analysis is based on applying a simple model of yield loss as a function of disease incidence across all scenarios, so the results should be interpreted in terms of relative loss and not in terms of actionable economic threshold values.

## Results

### Environmental vector pressure and weather conditions

**Figure 2:**
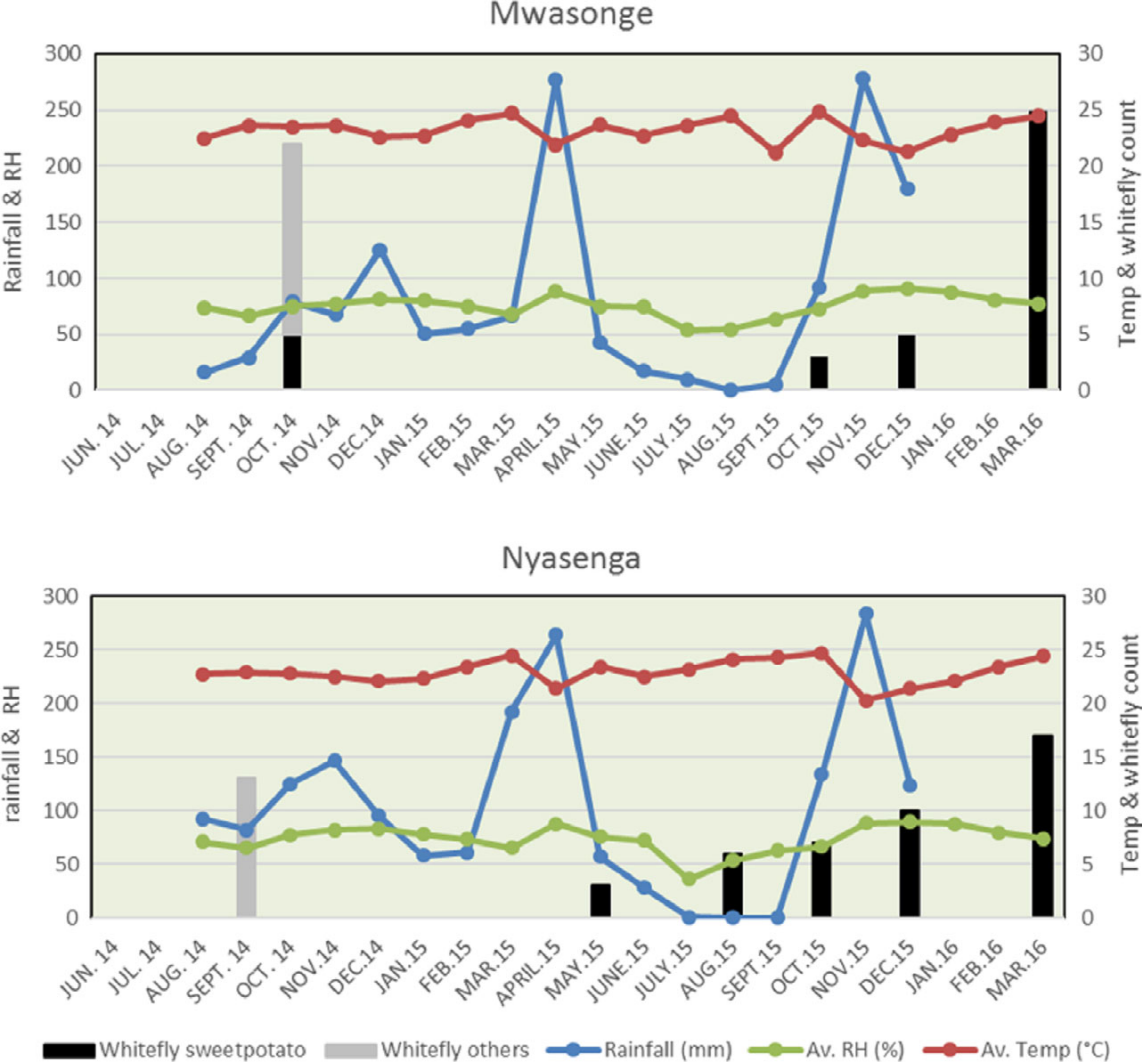
Weather conditions and whitefly counts during the experiments at the two trial sites.

Only a few (<10) volunteer sweet potato plants were found within a 250 m radius of the two sites at any of the sampling times between June 2014 and March 2016. Cultivated crops within a 250 m radius of the sweet potato seed plots included cabbage, okra, capsicum, spinach, watermelon, tomatoes, cucumber, amaranth, maize, rice and beans at the Mwasonge site, and pumpkin, beans, maize, rice, cassava, amaranth, cotton, cabbage and spinach at the Nyasenga site. Weeds were also present, but none were *Ipomoea* spp. There was minimal whitefly presence in the sweet potato plots and surrounding crops during most of the experiment. A spike in whitefly populations was seen in October 2014 in surrounding crops at both sites, principally in pumpkins and beans. The population of whiteflies at both sites started to increase in August 2015, this time principally in sweet potato (Fig. 2). Aphids were only observed in October 2014 at Mwasonge on cucumbers and beans (data not shown).

### Virus incidence

#### Viruses present in the environment at initiation of experiment

Results from small RNA sequence assembly analysis of bulked samples from nearby sweet potato fields at the time of installation of the experiment revealed the following viruses were present in the environment: SPFMV, SPVC, SPCSV-EA strain, SPLCV, sweet potato pakakuy virus and sweet potato symptomless mastrevirus 1. Because the latter two viruses are not known to cause any disease in sweet potato and occur only at extremely low titres in plants (J. Kreuze, unpublished data, International Potato Center, Lima, Peru), only SPFMV, SPVC, SPCSV and begomoviruses were considered relevant for this study.

#### Viruses in samples collected from the experimental plots

**Figure 3:**
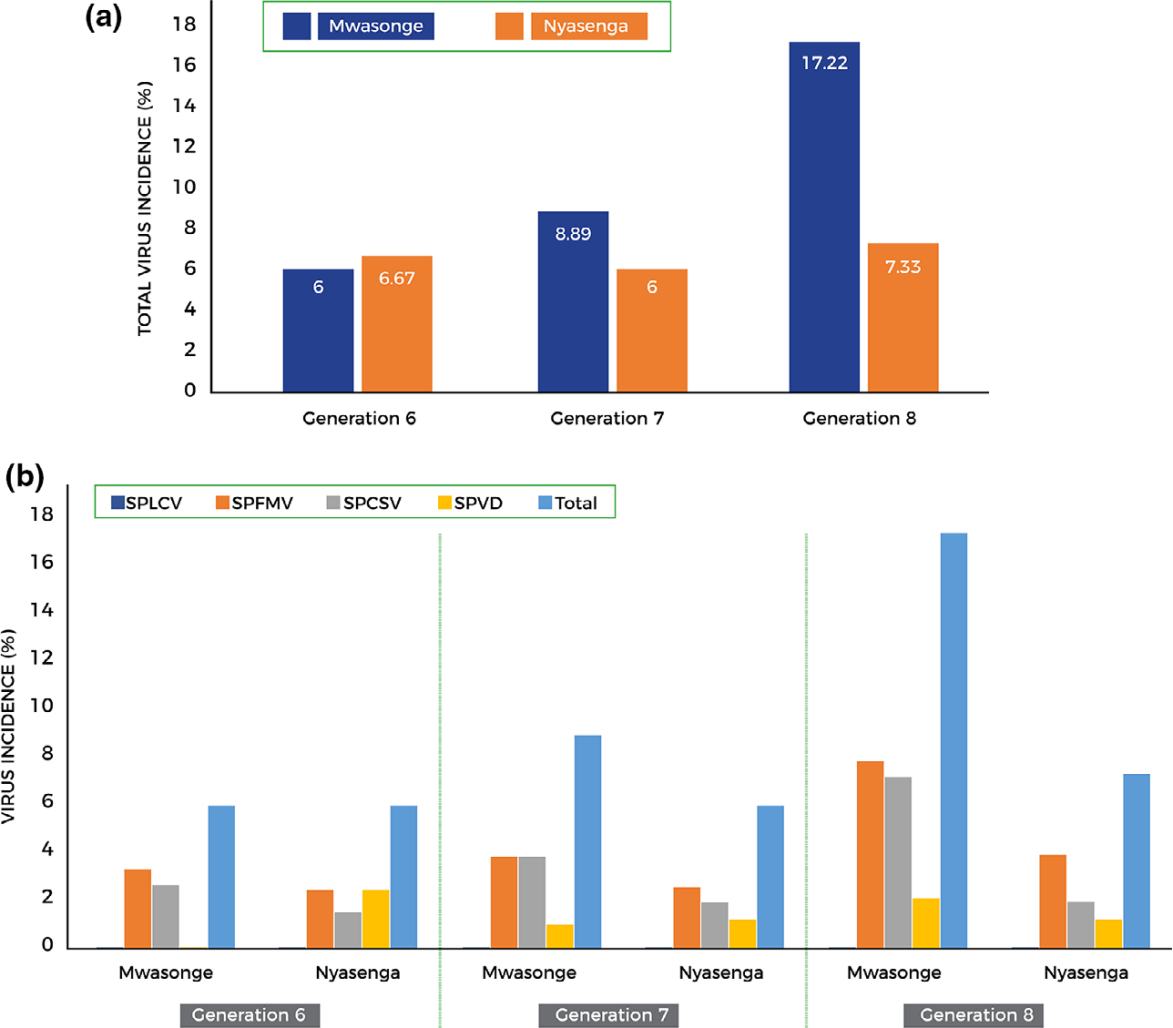
Virus incidence as detected by PCR assays at the high virus pressure area (Mwasonge) and the low virus pressure area (Nyasenga). (a) Total virus incidence, (b) incidence of specific viruses. Virus incidence was zero in the first five generations.

For the first five generations, all samples collected from vines maintained in net tunnels (Net-tunnel), vines harvested from the net tunnels and planted in the open field for one generation (Net-tunnel-OF) and vines grown in the open throughout (Open field (control)) tested negative for viruses. Virus infection started occurring in generation 6 and increased with successive generations at both sites (Fig. 3). There was higher virus incidence at Mwasonge as compared to Nyasenga. Only marginal changes in virus incidence occurred at Nyasenga while a sequential increase was noted at Mwasonge from generation 6 to 8. Viruses detected at the two sites were SPFMV and SPCSV occurring singly or coinfecting to form SPVD (Fig. 3b). SPFMV was the most prevalent at both sites.

**Figure 4:**
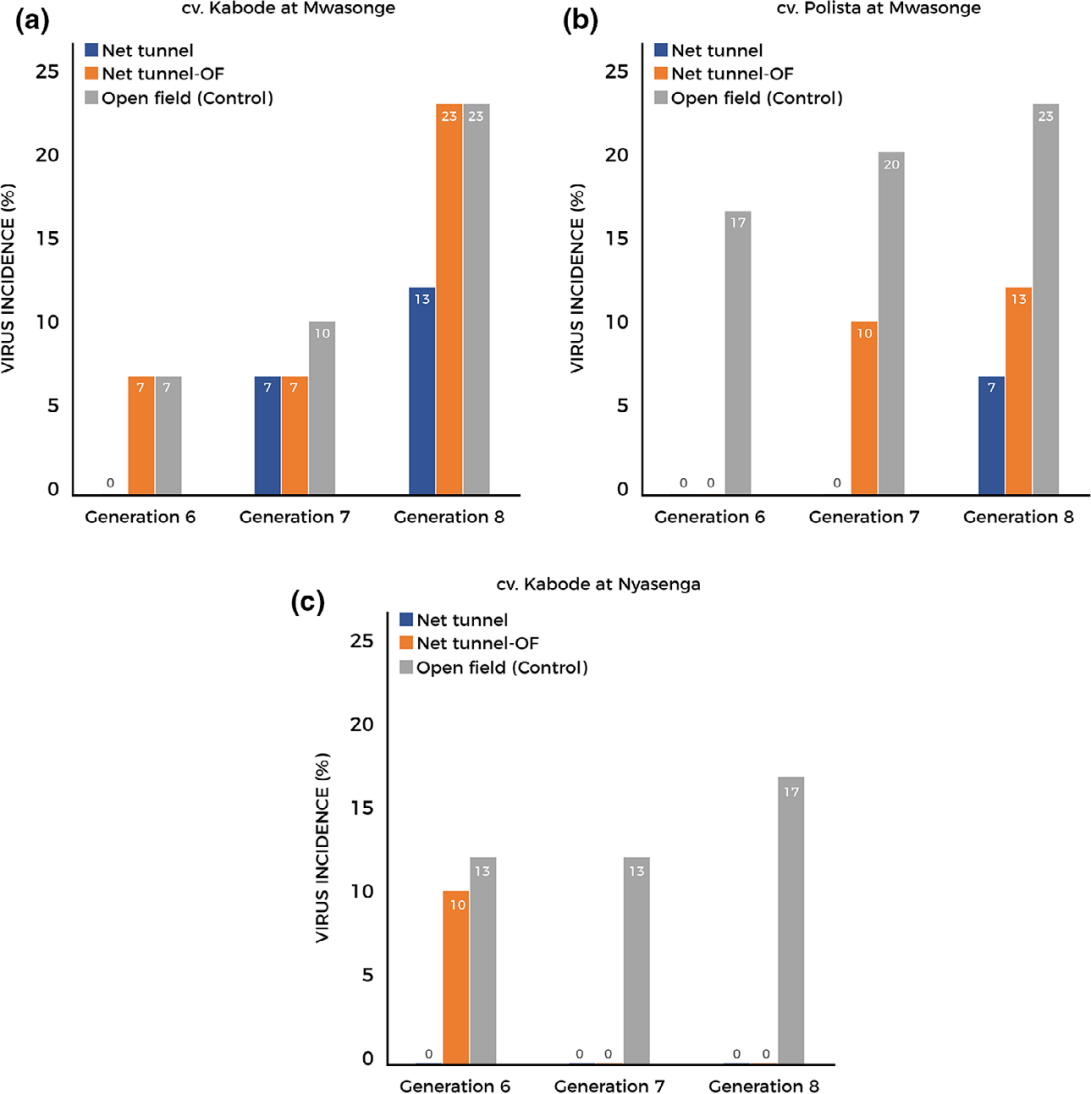
Incidence of viruses detected by PCR assays in sweet potato cv. Kabode and cv. Polista during the last three generations.

Vines of variety Kabode maintained in the net tunnels at Mwasonge had 7% and 13% virus incidence during generations 7 and 8, respectively (Fig. 4a). Vines of the same variety continuously grown at the open field (control) at Mwasonge had 7%, 10% and 23% virus incidence during generations 6, 7 and 8, respectively. Vines of the variety Polista maintained in the net tunnel at the high virus pressure area were diagnosed with 7% virus incidence in generation 8. Planting material of the same variety continuously grown at the open field had the highest virus incidence (Fig. 4b). Virus incidence was zero for variety Polista at the Nyasenga site (low virus pressure area) for the entire period of study, both for open fields and net tunnels. Vines of cv. Kabode grown in the net tunnel at this site were not infected by viruses throughout the study. On the other hand, vines of the same variety grown continuously in open fields at the same site had 13%, 13% and 17% virus incidence during generations 6, 7 and 8, respectively (Fig. 4c).

### Seed degeneration modeling

**Figure 5:**
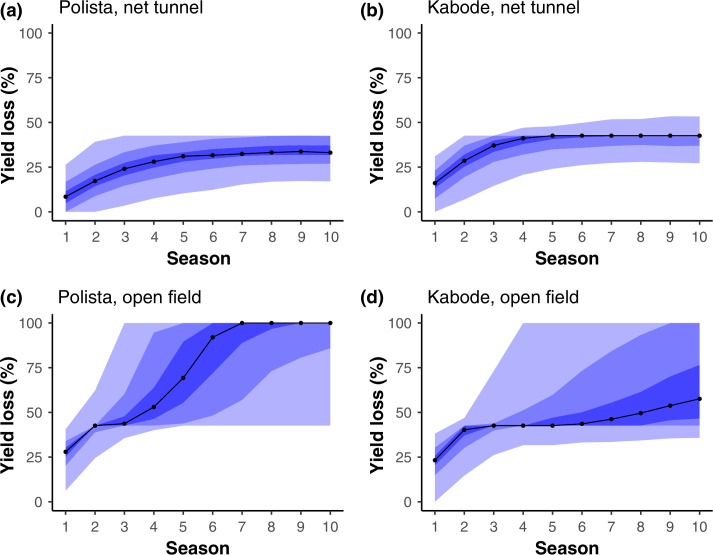
Modelled percentages of yield loss in sweet potato cv. Kabode and cv. Polista over 10 seasons in scenario analyses for the high virus pressure site (Mwasonge), in extrapolations based on estimates of infection rates from the experiment combined with a generic model of yield loss. The yield loss percentages are the results of 1000 simulations, where each simulation started with seed with no infection so that the initial infections came from the surrounding area (Thomas-Sharma *et al*., 2017). Black lines indicate the median (0.50 quantile) yield loss across the 1000 simulations, and the shading indicates quantiles starting at 0.05, with 0.25, 0.40, 0.60, 0.75 and 0.95 indicated.

Results from seed degeneration modelling illustrated how, for both Kabode and Polista, yield degeneration was reduced by the maintenance of vines under net tunnel conditions for each growing cycle, when compared to treatments where seed vines were grown in the open field (Fig. 5). This result was somewhat more pronounced for the cream-fleshed variety, Polista. The time series of likely degeneration based on these observations, in a scenario with a generic yield loss model, shows how a potential economic threshold such as 40%, would not be reached in net tunnels for many seasons, while the same threshold might be crossed in the open field after only a couple seasons.

## Discussion

Growing sweet potato seed in net tunnels reduced degeneration by limiting virus incidence. Previous on-station research had shown that well-managed net tunnels could be used to maintain virus-free vines for at least 33 months (Schulte-Geldermann *et al*., 2012). The insect-proof net tunnels block virus vectors (whiteflies and aphids) from accessing the plants. By avoiding physical contact between vectors and sweet potato plants, the crop is protected from virus infection. As indicated in this study, up to 100% protection can be achieved in low virus pressure areas. High degeneration rates in open fields is linked to exposure to vectors that transmit viruses from diseased sweet potato plants or wild *Ipomoea* spp. to newly established virus-free plants. An increase in virus incidence coincided with increased whitefly population. The population of whiteflies at both sites started to increase in August 2015, corresponding with the increasing virus incidence during the last three cycles of growth. The whiteflies were predominantly on sweet potato plants being established in neighbouring fields, therefore providing a favourable host for reproduction and multiplication of the vectors.

Generations 1 to 5, with zero virus incidence observed, experienced a period of limited rainfall leading to low cultivation of sweet potato in areas surrounding the experimental sites and therefore reduction in external disease inoculum. A favourable environment is very critical for plant disease manifestation in addition to pathogen–host interactions (Gergerich & Dolja, 2006). Sufficient rainfall and high humidity favour build-up of sweet potato virus inoculum due to increased cultivation, leading to increased infection rate and rapid deterioration. Weather conditions and disease pressure are therefore important considerations in management of sweet potato viruses and selection of sites for seed production. Thiele (1999) linked better seed potato production at higher altitudes to favourable temperatures and low disease pressure. Results of this study confirmed that Mwasonge village was a higher virus pressure area compared to Nyasenga. The Mwasonge site was by the shores of Lake Victoria where sweet potato is usually intensely grown due to favourable conditions.

That only SPCSV and SPFMV were detected in the study area reflects previous reports that these two are the most common and important yield-limiting sweet potato viruses in East Africa, especially when occurring together (Ndunguru *et al*., 2009; Adikini *et al*., 2016). High prevalence of SPFMV is consistent with findings by Clark *et al*. (2011) and Tairo *et al*. (2004) who reported the symptomless potyvirus to be the most common single infection virus at the Lake Zone, Tanzania. Efforts employed to control viruses in sweet potato should consider regional variability of the most significant viruses. This can aid in prioritizing resources, especially in diagnostics. Virus testing has become a major component of clean systems but is limited by high costs, especially when using the more sensitive molecular techniques (Boonham *et al*., 2014). Focusing on the most important viruses in a country can reduce the costs, therefore making clean seed more affordable to farmers. Differential susceptibility to viruses between cultivars such as that seen in this study between cv. Kabode and cv. Polista is another important consideration in management. Yield losses resulting from single infections in particular varies with variety and virus involved (McEwan, 2016). A susceptible variety like Beauregard has been reported to be reinfected quickly, leading to 80–90% yield losses within a single season in Uganda (Adikini *et al*., 2015) and up to 40% yield losses in five seasons in the USA (Bryan *et al*., 2003). On the other hand, landraces cultivated in areas where SPVD is prevalent are more resistant (Bua *et al*., 2009). In addition, some varieties have the ability to revert to virus-free status after several seasons of cultivation (Gibson & Kreuze, 2015).

Evaluation of the field data in the model from Thomas-Sharma *et al*. (2017) enabled consideration of the potential optimal time to seed replacement for combinations of variety and management with net tunnels. For some treatment combinations, there was not enough data available to generate estimates. Varieties Kabode and Polista were compared, with and without net tunnels, in an analysis with simplifying assumptions about the relationship between disease incidence and yield. Yield in the scenario analysis is sensitive to changes in management such as roguing rates and positive selection (Thomas-Sharma *et al*., 2017). Actionable economic thresholds can also be difficult to formulate without good models of yield loss and good information about farmer willingness to pay. In sub-Saharan Africa, the challenge of anticipating likely economic thresholds is compounded by socio-cultural values that surround sweet potato seed acquisition (Almekinders *et al*., 2019). For the farmers in this study, decline in cultivar performance might be tolerated for many seasons due to the subsistence nature of their farming systems. Limited record keeping makes it difficult to notice yield losses and therefore farmers may replace seed only at very high infection levels when the crop cannot produce any bulked roots. However, as farmers have more information available and more options, they may choose to use a lower economic threshold to get an economic return from higher quality seed. It is also important to note that in this experiment there was greater attention to removing inoculum sources than is likely on most farms in the region. Thus, the typical farmer in the region may experience substantially faster seed degeneration, motivating faster seed replacement.

This research provides evidence supporting use of insect-proof net tunnels among farmer-multipliers to reduce seed degeneration in sweet potato. Given their affordability in both construction and management, they can contribute greatly in the improvement of local seed systems. However, the net tunnels should be combined with other on-farm management options such as positive selection and roguing. Piloting in Ethiopia, Kenya, Mozambique, Nigeria, Rwanda, Tanzania and Uganda has shown that well-resourced, trained farmers are better adopters of the technology, therefore recommending it for basic seed production especially in high virus pressure areas (Ogero *et al*., 2017). This research also sheds more light on parameters to consider in seed degeneration studies, especially for vegetative crops. The need to consider variety, environmental virus pressure, weather conditions and agronomic practices in seed degeneration modelling was illustrated. This is consistent with examples from Thomas-Sharma *et al*. (2016) and Bryan *et al*. (2003). Seed degeneration models can help to inform farmers, and those who advise farmers, about the time to economic seed replacement. To stay in business farmers should operate at an equilibrium whereby cost of production equals revenue. Because cost of production includes other inputs, a farmer might decide to forgo or reduce investments in one input to compensate for the losses associated with the quality of seed. This implies that a farmer is likely to purchase quality seed when yield losses without the use of clean seed are greater than the cost of seed, all other factors constant. Use of net tunnels for sweet potato seed production might increase the cost of seed due to the additional investment. To keep the cost affordable, farmer-multipliers using the net tunnels are advised to sell after two rounds of open field multiplication thereby increasing the quantities.

## Supporting Information

**Table S1.** Parameters used in seed degeneration risk assessment.
