# Supplementary material for: Efficiency of insect-proof net tunnels in reducing virus-related seed degeneration in sweet potato
Source: Plant Pathol. 2019 Jul 22;68(8):1472–80. doi: 10.1111/ppa.13069 (PMC7198129; doi:10.1111/ppa.13069)
Supplement: Supplementary file 1 [file PPA-2019-PPA-13069-s1.docx]

**Table S1. Parameters used in seed degeneration risk assessment.**

These parameters are from the model of seed degeneration presented in Thomas-Sharma et al. (2017), which provides more details about each parameter and its role in the model. The parameter values used in the comparison of four scenarios from the current study (the four columns to the right of the table) were estimated based on the observed change in incidence for these scenarios. Four parameters multiplied together in the model (βWHM) were estimated as a single unit here for simplicity.

| **Para-meter** | **Description** | **Biological meaning of values** | **Default values used in Thomas-Sharma et al 2017** | **1. Variety Kabode, Open Field** | **2. Variety Kabode, Net Tunnel** | **3. Variety Polista, Open Field** | **4. Variety Polista,**  **Net Tunnel** |
| --- | --- | --- | --- | --- | --- | --- | --- |
| pHS_0_ | Initial proportion of healthy seed | 1=no seed infected  0=all seed infected | 0.8 (low starting infection scenarios)  0.2 (high starting infection scenarios) | 1 | 1 | 1 | 1 |
| K | Initial plant population (number) | Population at beginning of season based on planting rate in a small field | 100 | 270 | 270 | 270 | 270 |
| E | External inoculum | Amount of host/non-host inoculum surrounding a field | 0 (absence of external inoculum)  30 (presence of external inoculum) | 30  “High virus” | 30 | 30 | 30 |
| β | Maximum transmission rate per season | Maximum rate of disease transmission during the season when there are no limiting factors for disease spread | 0.02 | Mean of βWHM = 0.0048  Standard deviation of βWHM = 0.0032 | Mean of βWHM =  0.0030  Standard deviation of βWHM = 0.0026 | Mean of βWHM =  0.0063  Standard deviation of βWHM =  0.0032 | Mean of βWHM =  0.0015  Standard deviation of βWHM =  0.0026 |
| W | Proportional change in infection due to environment | W=1, maximally conducive environmental conditions  W=0, environmental conditions that do not support transmission | 0.8 (highly disease-conducive weather)  0.2 (marginally disease-conducive weather) |  |  |  |  |
| H^1^ | Proportional change in infection due to host genetic resistance | H=1, highly susceptible  H=0, immune | 1 |  |  |  |  |
| M^1^ | Proportional change in infection rate due to vector management | M=1, indicates no management  M=0, indicates vector or pathogen eradication | 1 |  |  |  |  |
| A^1^ | Proportion diseased plants remaining after roguing | A=1, indicates no roguing  A=0, indicates all diseased plants removed | 1 | 0.5  There was roguing, everywhere- kept constant | 0.5 | 0.5 | 0.5 |
| G | Seed production rate in healthy plants | Number of seed produced per healthy plant | 4 | 2 | 2 | 2 | 2 |
| Z^1^ | Proportional selection against diseased plants (through positive or negative selection) | Z=1, indicates no seed selection  Z<1, indicates proportional selection against diseased plants  Z=0, indicates complete selection against diseased plants | 1 | 0.7 | 0.7 | 0.7 | 0.7 |
| C | Indicates differential seed production in the diseased plants as a proportion of seed production in healthy plants | C=0, indicates no seed production in diseased plants  C=1, indicates no difference in seed production between healthy and diseased plants  C<1, indicates reduced seed production in diseased plants  C>1, indicates increased seed production in diseased plants | 0.9 | 0.9 | 0.9 | 0.9 | 0.9 |
| R | Reversion rate | Proportion of diseased plants that produce disease-free seed | 0.1 | 0.05  constant and small | 0.05  constant and small | 0.05  constant and small | 0.05  constant and small |
| Φ | Proportion certified (or otherwise completely disease-free) seed purchased | ϕ=1, all certified seed  ϕ=0, no certified seed | 0 | 0 | 0 | 0 | 0 |
| Θ | Rate of decline of yield (at end of season) as a function of disease incidence | 0<θ≤0.5, indicates yield decline low for low disease incidence, then increases  θ=negative, indicates yield decline is rapid for low disease incidence, then slows  θ=0, indicates constant rate of decline | 0.2 | 0.2 | 0.2 | 0.2 | 0.2 |
| γ | Proportional change in effect of disease incidence on yield loss for late season versus early season | γ=0, indicates no yield loss due to late season disease incidence  γ=1, indicates no difference between early and late season effects of disease incidence on yield loss | Not used in general models |  |  |  |  |
| minY | Minimum yield | Units of yield produced by a severely infected plant | 0 | 0 | 0 | 0 | 0 |
| maxY | Maximum yield | Units of yield produced by a healthy plant | 100 | 100 | 100 | 100 | 100 |
